# Short-Term Outcome of Foam Sclerotherapy With Sodium Tetradecyl Sulphate for the Treatment of Venous Malformation in Dhaka, Bangladesh: A Single-Centre Experience

**DOI:** 10.7759/cureus.99251

**Published:** 2025-12-15

**Authors:** Afroza Akter, Masrur Akbar Khan, Md. Arif Uddin Khan, Nur Mohammod Sayed Bin Aziz, Md. Mezbahur Rahman

**Affiliations:** 1 Infectious Diseases Division, International Centre for Diarrhoeal Disease Research, Bangladesh (icddr,b), Dhaka, BGD; 2 Vascular Surgery, National Institute of Cardiovascular Diseases, Dhaka, BGD

**Keywords:** bangladesh, sclerotherapy, sodium tetradecyl sulphate, vascular surgery, venous malformation

## Abstract

Background: Treatment of venous malformations is challenging for physicians. Previously, surgery was regarded as the only treatment option. Recently, sclerotherapy has been proven to be a low-cost, effective, and convenient substitute with minimal discomfort and blood loss. It doesn’t require local anaesthesia or postoperative care, allowing for immediate return to daily activities. This paper objectives to account for a single-centre experience with the use of foam sclerotherapy using sodium tetradecyl sulfate (STS) for venous malformations and to present the short-term clinical outcome, including symptom improvement, safety, and size reduction.

Methods: A prospective study was conducted in the Vascular Surgery Department, Bangladesh Medical University (BMU) between July 2021 to December 2021. We selected 21 patients with low-flow venous malformations confirmed by magnetic resonance imaging (MRI) and duplex ultrasonography. All the patients received STS foam sclerotherapy after clinical and radiological assessment. Each lesion received multiple sessions. A follow-up was done for radiological and clinical improvement of the lesions.

Result: The median age of the participant was 17 (12, 29) years. Around half of the participants (n=9; 42.9%) were from the 11-20 years of age group, with almost a similar sex distribution. Both upper and lower limbs were the most commonly affected regions (n=9, 42.9%; in each limb). Swelling was observed in all participants, and 16 presented with pain. After sclerotherapy, swelling and pain decreased in all participants (100%). A total of nine (42.9%) participants required three sessions of sclerotherapy, followed by eight (37.2%) patients who required four sessions. Several sessions of 3-4 ml of STS were required by 38.1% (n=8) participants, followed by 33% (n=7) requiring 2-2.9 ml/session. All participants experienced pain following sclerotherapy. While 14 (66.7%) of the participants obtained ≥50% response, among them five (23.8%) were completely reduced. The other seven (33.3%) cases resulted in <50% resolution.

Conclusion: Overall, STS sclerotherapy is an effective treatment procedure for low-flow venous malformations with a low complication rate. Moreover, it offers a convenient and cost-effective outpatient-based treatment option to most patients with uncomplicated venous malformations.

## Introduction

Venous malformation (VM) is the most common type of congenital vascular malformation (CVM), with an incidence of one to two per 10,000 births and a prevalence of 1% [1]. Despite their presence since birth, they might not become clinically evident until later life. Sometimes initiated by events like hormonal changes during puberty, infection, or injury, more often without any sign of spontaneous regression [2]. Histologically, they are made up of dormant endothelium-lined abnormal vascular channels [3]. VMs sometimes result in stagnant blood flow, ultimately predisposing to spontaneous thrombosis, which in turn might lead to serious complications, both local and systemic. The resultant clinical symptoms are pain, swelling, changes in the overlying skin, and exaggerated growth of tissue and limb [4].

VMs with abundant vascular or lymphatic spaces benefit from sclerotherapy than from cellular matrix. Pathognomonic ultrasound features of VMs include phleboliths, which are detected in approximately 20% of cases [5]. However, magnetic resonance imaging (MRI) remains the preferred imaging modality for pre-procedure VM diagnosis to support optimal management planning and is also used for post-procedure evaluation of the lesion. MRI is also a better tool to evaluate clinical outcomes after sclerotherapy. To ascertain the diagnosis, a correlation of clinical with Doppler ultrasound and MRI findings should be made [6].

Low-flow vascular malformations include VM, lymphatic malformations, capillary, and complex-combined malformations, but without any high-flow arterial component. The various management options for low-flow vascular malformation include percutaneous sclerotherapy, surgery, elastic compression, and laser therapy. Sclerotherapy with or without surgery comprises the treatment options for the VMs. When the lesion was deemed completely resectable without detrimental anatomic and functional disabilities, surgery was regarded as the primary treatment option in traditional practice. However, surgical treatment can often culminate in a loss of motor function, nerve damage, and massive bleeding when the lesion is more extensive [7]. Extreme difficulty in controlling haemorrhage during excision of complex lesions has led physicians to adopt sclerotherapy as the favoured approach. Sclerosing agents act by inducing endothelial damage of blood vessels, thereby producing fibrosis and thrombosis. They remain particularly beneficial for vascular malformations without marginal drainage and lesions of small vascular beds with weak or absent flow. Several sclerosants, such as absolute alcohol, 1% polidocanol, hypertonic saline solution, sodium tetradecyl sulphate (STS), and sodium morrhuate have been used for this purpose [8]. Sclerosing agents are viable alternatives to surgery with lower toxicities. Bleomycin produces a sclerosing effect on endothelial cells, though it is an anti-tumour agent that inhibits the synthesis of deoxyribonucleic acid (DNA). Bleomycin is typically used as the first-line treatment for head and neck VMs [1]. Sodium tetradecyl sulfate is a synthetic, surface-active substance first described in 1946 [9]. STS is a widely used sclerosing agent that is relatively effective, painless, and incite desired endothelial damage. Sclerosis with foam technique can be performed as an outpatient procedure, without any anaesthesia [10]. Thus, sclerotherapy with STS is advised in terms of lower adverse effects [11]. However, STS causes epidermal necrosis that may occur with extravasation of concentrations higher than 1%. Allergic reactions and post-sclerotherapy hyperpigmentation may also occur [9].

The number and nature of VM differ from one country to another. However, only surgical excision has a limited role in most cases due to the functional and cosmetic problems. Minimal discomfort, negligible blood loss, and low cost are the advantages of sclerotherapy. There is no obligation for local anaesthesia or postoperative management. The patient can continue his daily activities immediately. STS has been widely used in our context with fewer side effects, easy availability, and ease of usage. Despite its extensive use by clinicians, to the best of our knowledge, no study has been conducted yet in Bangladesh regarding the short-term outcome of this commonly used sclerosing agent in VMs. The objective of this study was to evaluate the short-term outcome, including symptom improvement, safety, and size reduction of this commonly used STS sclerotherapy as the treatment of VM.

## Materials and methods

Study site and ethical considerations

This prospective study was conducted from July 2021 to December 2021 in the Vascular Surgery Department at Bangladesh Medical University (BMU), Dhaka, Bangladesh. Ethical approval was obtained from the BMU Institutional Review Board (Approval No. BSMMU/2021/954). All patients enrolled in this study were informed about the objective and procedure of the study. Informed written consent was taken from all participants or participants' guardians. Assent was obtained from the participants aged 11 to 17 years.

Study participants

The study participants visited the vascular surgical outpatient department for treatment of an uncomplicated low-flow cutaneous VM. Low-flow VMs were confirmed by MRI and duplex ultrasonography before inclusion in the study. We excluded the participants who were known cases of allergy to STS sclerotherapy, had previously failed attempts of any sclerotherapy, complicated cutaneous VM, or patients who had not consented to STS. Demographic profile, including age and sex, site of lesion, symptoms duration, volume of STS, and number of sittings required, complications, and outcome of the procedure, was recorded.

STS foam preparation

We used the Tessari technique for STS foam preparation to produce a stable microfoam through rapid mixing of STS solution with air in a dual-syringe system connected by a three-way stopcock [12]. Uniform microbubbles were generated that enhance sclerosant-endothelium contact [12].

Imaging measurement methods

The lesion size was calculated in centimetres (cm) by multiplying the largest width and height of each lesion to capture the complete morphology of the malformation. VMs were evaluated using Doppler ultrasound and MRI. Doppler ultrasound provided a real-time assessment of lesion morphology and flow characteristics. MRI was performed for tissue involvement and two-dimensional measurements. Together, these modalities enabled accurate detection of lesion site, anatomical mapping, and size estimation.

Procedures

Before each sclerotherapy injection, a clinical evaluation was conducted on all patients. The 3% STS injection was given after painting and draping of the epicentre of the lesion using an insulin syringe. First, it was confirmed that there was no free blood backflow by withdrawing the plunger of the syringe. Then, STS sclerotherapy was injected at multiple sites in the lesions. All the patients were advised to take an oral analgesic after the procedure for three days. The treatment was repeated if there was an absent response or an incomplete response. Four interventions were provided utmost. The outcome of the procedure was noted against the number of interventions by STS. A pressure bandage was applied for 24 hours, and participants were requested to remove the bandage their own at home.

Session intervals

Participants were advised to visit the outpatient department after three weeks of enrollment in the study. During the follow-up time, an MRI and Doppler ultrasound were done for every case to measure the reduction in VM size.

Sample size

The sample size was determined based on the primary outcome variable, complete reduction of lesion size (yes/no). Since the outcome was categorical and expressed as proportions, the calculation was performed using a two-sided test with a 5% level of significance and 80% power. A previously reported cure rate of 46% [1] was considered the reference proportion, while the expected proportion of complete reduction in the current study was hypothesised to be 75%, representing a clinically meaningful difference of 29%. Using standard parameters (Zα/2 = 1.96 and Zβ = 0.84), the required sample size was calculated to be 21. Therefore, a total of 21 patients who met the inclusion and exclusion criteria were selected as the study sample.

Statistical analysis

We used a standard semi-structured data collection sheet to collect the necessary information from the study participants. In this study, the intervention response was classified based on the degree of reduction in malformation size from baseline. A reduction of ≥50% of the initial size was considered a ‘fair response’, while a reduction of <50% was categorised as a ‘poor response’ during the final follow-up. Statistical analysis was conducted using “Stata” version 13.0 for Windows software (StataCorp LLC, College Station, TX, USA). All data were presented in mean values +/- standard deviation. The results were presented in tables and figures. Fisher’s Exact Test was used to determine the significant association between two categorical variables. Results were recorded as statistically significant if a p-value <0.05.

## Results

Distribution of study participants

The median age of the study participants was 17 (12, 29) years. Most of the participants (42.9%) were from the 11-20 years of age group, followed by 23.7%, 14.3%, 14.3%, and 4.8% from the <10 years, 21-30 years, 31-40 years, and >40 years of age groups, respectively. Twelve (57.1%) of the study participants were female subjects, while the rest were male (42.9%) (Table 1).

**Table 1:** Distribution of study participants according to age and sex (n=21) Data were expressed as frequency and percentage

Variables	Value
Median age (interquartile range)	17 (12, 29) years
Age	
<10 years	5 (23.7)
11-20 years	9 (42.9)
21-30 years	3 (14.3)
31-40 years	3 (14.3)
>40 years	1 (4.8)
Gender	
Female	12 (57.1)
Male	9 (42.9)

Distribution of study participants according to VM characteristics

Most of the VMs were located in the limbs. Both the upper and lower limbs had equal numbers of lesions. Nine (42.9%) patients had their lesions in their upper limbs, with a similar number in their lower limbs, followed by two (9.5%) in the head-neck region and only one in the trunk (4.7%), respectively. Mean size of the VMs in different regions includes 58.81±43.18 cm^2^ in lower limbs, followed by 23.75±22.91 cm^2^, 21±26.87 cm^2^ & 8.14 cm^2^ in upper limbs, head-neck region, and trunk, respectively (Table 2).

**Table 2:** Distribution of study participants according to anatomical location and mean size of the venous malformation SD = standard deviation Data were expressed as frequency, percentage, and mean ± SD

Site	No. of study participants (%)	Mean size of the VM (±SD) cm^2^
			Upper limb	9 (42.9)	23.75 (±22.91)
Lower limb	9 (42.9)	58.81 (±43.18)
Trunk	1 (4.7)	8.14 (±0)
Head neck	2 (9.5)	21 (±26.87)
Total	n=21	37.77 (±36.76)

Pre-procedure presentation and post-procedure changes in clinical symptoms

All participants presented with swelling, while 16 presented with pain, 14 with cosmetic blemish, nine with restricted joint movement, three with bleeding, and two with ulceration. After sclerotherapy, swelling and pain decreased in all participants. Most of the symptoms improved, including movement of joints, bleeding, and ulceration. While only half of the participants with cosmetic blemishes had improved outcomes, none of the participants experienced worsening of symptoms (Table 3).

**Table 3:** Pre-procedure presentation and post-procedure changes in clinical symptoms Data were expressed as frequency and percentage

Clinical symptoms	Pre-procedure	Post procedure	
		Improved cases (%)	Not improved cases (%)
Swelling	21	21 (100%)	0 (0%)
Pain	16	16 (100%)	0 (0%)
Restricted movement of joints	9	7 (77.78%)	2 (22.22%)
Bleeding	3	2 (66.67%)	1 (33.33%)
Ulceration	2	1 (50.0%)	1 (50%)
Cosmetic blemish	14	7 (50.0%)	7 (50.0%)

Distribution of study participants according to sclerotherapy details

Nine (42.9%) of the participants had three sessions of sclerotherapy, with five of them receiving 2-2.9 ml of drug per session in the present study. On the contrary, eight (38.1%) had four sessions, with five of them receiving 3-4 ml of drug per session. Only four (19%) had two sessions, with three of them receiving <2 ml of sclerosant (Table 4).

**Table 4:** Distribution of the study participants according to the number of sclerotherapy sessions and doses of sclerosant injected during the procedure Data were expressed as frequency and percentage

No. of sessions	Doses of sclerosant injected				Total
	<2 ml	2-2.9 ml	3-4 ml	>4 ml	
2	3	1	0	0	4 (19.0%)
3	1	5	3	0	9 (42.9%)
4	0	1	5	2	8 (38.1%)
Total	4	7	8	2	n=21 (100%)

Distribution of study participants according to complications of sclerotherapy

**Figure 1:**
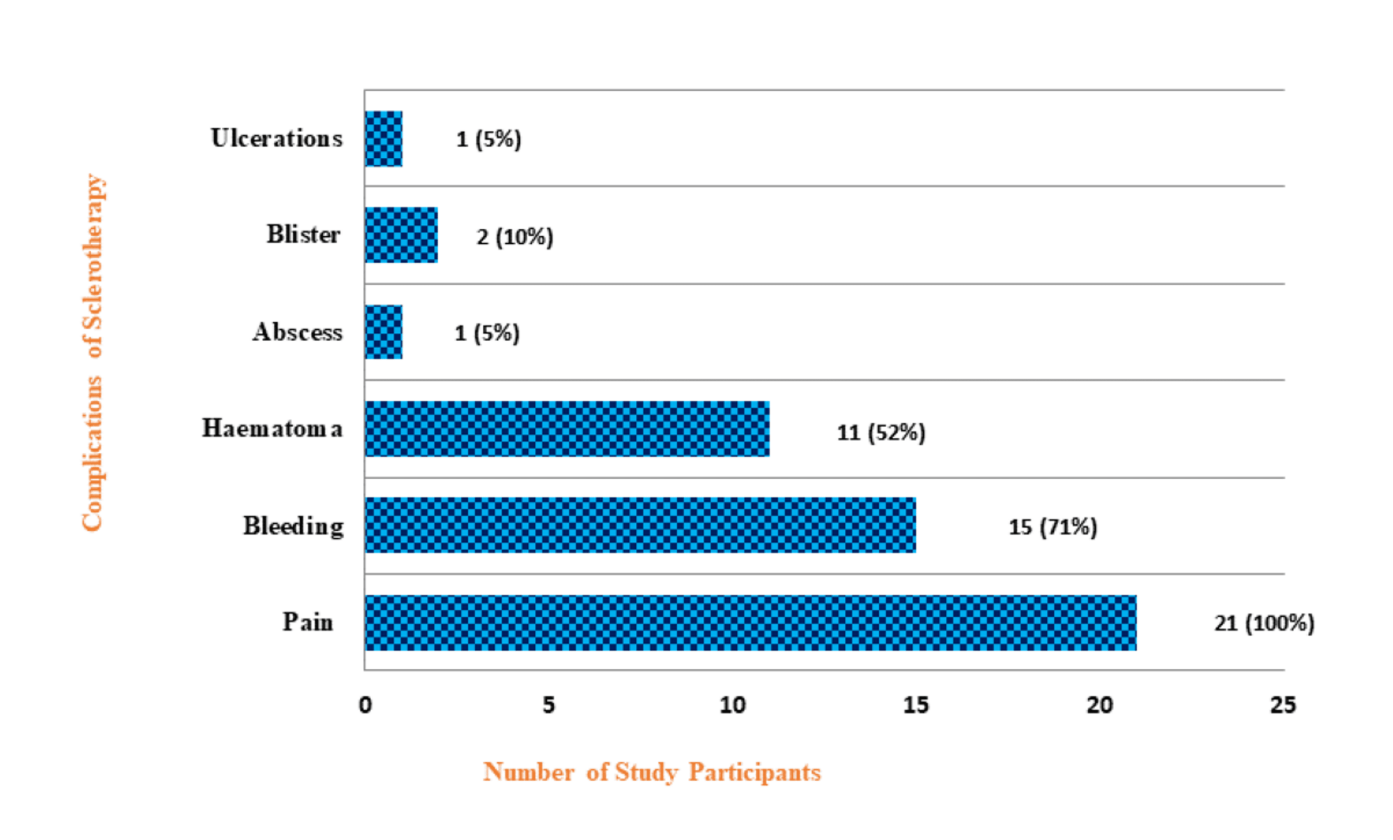
Distribution of the study participants according to the complications of sclerotherapy

All participants (100%) experienced pain during sclerotherapy. Fifteen (71%) participants had bleeding, while 11 (52%) experienced haematoma, followed by two (10%) blister, one (5%) ulceration, and one (5%) abscess, respectively, after sclerotherapy (Figure 1).

Association of characteristics of the lesion to clinical response and sclerotherapy details with clinical response

Among participants with VMs in the upper extremity, most (n=8) demonstrated a fair response to sclerotherapy, while only one showed a poor response. In contrast, among those with lower extremity lesions, more than half (n=5) exhibited a fair response, and four had a poor response. Four participants who underwent two sessions of sclerotherapy demonstrated a fair response. Similarly, six of nine participants who received three sessions and four of eight who underwent four sessions showed a fair response. With respect to the dose of sclerosant used, all four participants who received less than 2 ml per session exhibited a fair response. Additionally, five of seven participants receiving 2-2.9 ml and five of eight receiving 3-4 ml per session showed a similar response. In contrast, both participants who received more than 4 ml per session demonstrated a poor response. All these differences were not statistically significant (Table 5).

**Table 5:** Characteristics of lesions, number of sclerotherapy sessions, and doses of sclerosant to clinical response (n=21) STS = sodium tetradecyl sulfate *Fisher’s exact test was done; p<0.05 was considered statistically significant

Variables		Response to STS foam sclerotherapy		p value^*^
		Fair response, n=14 (≥50% reduction)	Poor response, n=7 (<50% reduction)	
Location of lesion	Upper extremity	8	1	0.168
	Lower extremity	5	4	
	Head & neck	1	1	
	Trunk	0	1	
Sclerotherapy sessions	2	4	0	0.292
	3	6	3	
	4	4	4	
Doses of sclerosant injected	<2 ml	4	0	0.146
	2-2.9 ml	5	2	
	3-4 ml	5	3	
	>4 ml	0	2	

## Discussion

The actual incidence of VMs in Bangladesh is unknown. This study was done in the Department of Vascular Surgery, BMU, among the participants with low-flow VM who received foam sclerotherapy with STS and were selected according to inclusion and exclusion criteria. The objective of this study was to evaluate the short-term outcome of STS sclerotherapy as the treatment of VMs. We found that interventions with STS sclerotherapy significantly improved the clinical symptoms and also reduced the size of VMs.

VM poses a difficult challenge for physicians in its satisfactory treatment. Based on the histological appearance of the abnormal channel, flow characteristics, and clinical behaviour, VMs can be classified into low‐flow and high‐flow vascular malformations [13]. Extreme difficulty in controlling haemorrhage during excision of complex VMs. This led physicians to adopt sclerotherapy as the favourable approach. The advent of sclerotherapy as a convenient, cheap, and minimally invasive procedure has prompted its use as mono-therapy or in conjunction with surgery [14]. Even though VMs are rarely associated with life-threatening problems, when noticed in the visible zone, and therefore, the patient becomes concerned for treatment as it affects the quality of life [15]. We used only 3% solution of STS foam sclerotherapy for the treatment of the VMs. The STS solution is not recommended to mix with other anaesthetic solutions or heparin because it will become turbid or form a new compound or become incompatible [9].

In this study, the median age of the participants was 17 years, and similar results were revealed in a study conducted in Pakistan [1]. However, in another case series of 13 patients, the mean age of 18.2 years and age range between eight months and 54 years was reported [16]. The male-female distribution of the participants with VMs in our study was almost similar to the ratio among males and females in another study [17].

All participants in our study presented with swelling along with pain, cosmetic blemish, restricted movement of the joint, bleeding, and ulceration. Accordingly, McCafferty and Jones mentioned that VMs generally cause functional or cosmetic symptoms related to size and location [5]. In addition, they commonly present with pain and swelling due to venous engorgement, localised thrombosis or thrombophlebitis, or local haemorrhage [18]. Similarly, van der Vleuten et al. reported that symptoms may vary and include cosmetic complaints, localised intravascular coagulopathy, pain, swelling, and functional limitations [18].

In this analysis, the upper and lower limbs were the most commonly affected region (42.9%), while the head and neck (9.5%) and trunk (4.7%) were the least commonly affected. Albeit their presence since birth, VMs often do not manifest until later childhood or puberty. They can happen anywhere in the body, but 40% occur in the head and neck region, 40% in the extremities, and 20% in the trunk [5]. However, in the case series by Ahmad, the most common site of VM was the head and neck, followed by the limbs and trunk [1].

The mean size of the lesions was 37.77±36.76 cm^2^. In contrast, previous studies reported smaller lesion sizes: one study observed a range of 0.48 to 5 cm² [19], while another reported 1 to 8 cm² [16]. The mean lesion size in the current study was much larger than that reported in earlier studies. These differences can be attributed to the presence of multiple lesions at a single site, which collectively increase the measured area. Moreover, variations in lesion shape across cases contribute to discrepancies in size between studies. The lesion size was calculated by multiplying the largest width and height of each lesion, whereas the measurement methods used in other studies were not clearly specified. Therefore, variability in lesion size across studies may also reflect differences in measurement techniques.

There are diverse perspectives and modalities for the use of sclerosing agent distribution in different studies. It depends on the size of the lesion and the maximum dose per session. In our study, nearly half (42.9%) of the participants required three sessions, followed by those (37.2%) requiring four sessions. We used 3-4 ml of sclerosants in over a third (38.1%) of the participants, followed by 2-2.9 ml in another third (33%). In a previous study, a protocol of 0.1 to 1 mL injection was used per session, requiring up to 10 sessions, which resulted in regression of malformation in all the cases [20]. Different studies suggested different volumes of STS dosages, like 4 mL of a 3% solution or up to 10 mL of a 1% solution for the treatment of VMs [9,21]. But our findings suggested that 2 to 4 mL of 3% STS is most effective for the VMs treatment. The number of interventions varied according to the lesion size. The interval between the injections was usually two to four weeks. It allows the induration and inflammatory reaction to subside [22]. For larger lesions, up to ten injections at two to four weeks' intervals were administered [20].

Pain was the most common complication after sclerotherapy experienced by all participants. Bleeding and hematoma were the next common complications after sclerotherapy. A study from Japan also reported that transient local pain, erythema, and swelling due to inflammatory reaction with phlebitis were seen after the procedures in almost all patients [23]. Similarly, according to another study, the side effects patients faced were pain and oedema after injection, with only 12% having mild superficial ulceration after STS injection, which healed without scarring [1].

In this study, all participants presenting with swelling and pain had improvement in symptoms following sclerotherapy. Other symptoms, like cosmetic blemishes, restricted joint movement, bleeding, and ulceration, also improved. While 14 (66.7%) of the participants obtained ≥50% response, among them five (23.8%) were completely reduced. The other seven (33.3%) cases resulted in <50% resolution. Another study showed that sclerotherapy with 3% STS given in 13 patients with venous malformations resulted in the lesions regressing by 90% to 100% in 11 cases after a mean of four injections, with no improvement in two cases [24]. O’Donovan et al. demonstrated that sclerosis therapy was beneficial in 86% of their treated patients with VMs [25].

Limitations

Several limitations are noted in our study. First, it was conducted on a relatively small number of VM cases. Findings thus obtained may not represent the whole picture. Second, only uncomplicated cases were involved in diverse regions, including upper and lower limbs, head and neck, and trunk, which might include minor procedural variability and preclude any detailed comparative analysis. Third, only short-term outcomes were measured. The outcomes may be different with long-term follow-up. Finally, this was a single-institution study that might not reflect the whole population. The findings of the study indicated that STD sclerotherapy is a useful, convenient, and safe intervention for the treatment of uncomplicated VMs. However, further study with a large sample size is needed to assess the optimal dose and number of sittings in order to achieve the optimum response.

## Conclusions

Sclerotherapy is the mainstay of treatment with absolute alcohol and sodium tetradecyl sulphate sclerotherapy (STS) is the most commonly used sclerosing agent and an effective treatment procedure for low-flow venous malformations with a low complication rate. This can be considered an excellent therapeutic success. Furthermore, it provides a convenient and affordable outpatient-based option for uncomplicated venous malformations. When a conservative approach is preferable, this modality of treatment offers an alternative to conventional methods like surgical excision. Surgical intervention is reserved for cases with residual post-sclerotherapy lesions or cosmetic concerns, while laser therapy is a useful option for superficial and oromucosal venous malformations. The advantage of STS is that it causes minimal discomfort with insignificant blood loss. It can be administered without local anesthesia without any specific post-procedure care. The patient can carry on his daily chores immediately. It can be considered as a safe, effective, and convenient option in all cases of uncomplicated cutaneous low-flow venous malformations.
